# Minute Anatomy of the Human Tooth

**Published:** 1883-10

**Authors:** Frank Abbott

**Affiliations:** New York


### MINUTE ANATOMY OF THE HUMAN TOOTH.

AN ADDRESS BEFORE THE DENTAL SOCIETY OF THE STATE OF NEW YORK,
BY FRANK ABBOTT, M. D., NEW YORK.

*Mr. President and Gentlemen :*

I suppose you will all recognize the fact that in order to treat the teeth or any other portion of the human frame rationally, you should first understand the absolute structure of the part itself. In fact it is thought by many that no one has any right to operate upon teeth, or treat any other part of the body, unless they understand the structure of the part upon which they are operating. I need not tell you that it is necessary to understand the structure of the teeth perfectly in order to be able to treat them in a manner to save them. I need not say to you that it is necessary to save teeth in order that patients may enjoy good health. You know it is impossible for a person's digestion to be in perfect order unless the primary digestive apparatus is in good healthy working condition. If the teeth are out of order the whole digestive track must necessarily be more or less affected. It cannot be otherwise. That being the case it stands us in hand to be careful in the study of this organ, that we may know just how, where and when to work. We see before us a diagram of an upper central incisor tooth, cut as nearly through the center as I could conveniently cut it, magnified a little over 10,000 diameters. This diagram I propose to explain to you as clearly as my knowledge of the subject will permit. First we have the crown,that portion which projects out of the gum; the neck, to which the gum is attached, and the root of the tooth, which stands in the socket formed by the alveolar process. The main portion of the tooth is composed, as you all know, of the dentine, or what was formerly called the ivory of the tooth. The crown is covered by the enamel, which is very much harder than the dentine, and put there for the purpose of withstanding the attrition of biting and mastication. This is covered, as you will observe, by a little epithelial layer. This membrane was discovered by Alexander Nasmyth in 1839, and is known as "Nasmyth's membrane." The root of the tooth is covered by a portion that is called the cementum, or *crusta-petrosa.* This more particularly resembles bone and contains more organic substance than dentine for its more perfect attachment to the periosteum, which covers the outside, or rather lines the socket in the alveolus, and is reflected upon the root of the tooth. The tooth was, up to 1678, I think, supposed to be a crystaline mass without any organic structure whatever. It was supposed to be a separate organ from the rest of the body, and simply stood in the socket without any other than mechanical attachment. The same, for instance, that holds a nail when it is driven into a piece of wood, and expressed by the word gomphosis—a word still used by some anatomists in this connection.

In 1678, a German, I think he was, by the name of Leeuwenhoek, discovered what he called "straight and transparent pipes" in the dentine in the teeth. He was the first who promulgated this doctrine. This man's name, therefore, we all should respect as that of the man who made the first recorded discovery of importance in the structure of these important organs. From that time up to 1839, this was the theory and understanding of the structure of the human tooth. The enamel was still supposed to be a crystaline mass upon the surface of the crown, without any organic structure whatever. The "straight and transparent pipes" of the dentine were supposed to be filled with a jelly-like substance. That is all that was known of the anatomy of teeth until the year 1839, when Alexander Nasmyth, an Englishman, published the results of his researches in this direction. In his published work he gives drawings, and describes a reticular structure of both the dentine and enamel; also what he calls a baccated appearance of the "pipes" of Leeuwenhoek, called by him, I believe, "tubules" or tubes. Mr. Nasmyth was as much at a loss, however, as to the nature of the contents of these tubuli as Leeuwenhoek, or any previous observer had been. In 1844, Dr. P. B. Goddard, of Philadelphia, wrote a work upon the "Anatomy, Physiology and Pathology of the Human Teeth," in which work he quotes nearly everybody who had written upon the subject before him. He states that the reticular structure and the contents of the tubuli are a gelatinous mass, which is moistened by fluid which comes out of the pulp and works its way off into the dentine. He says nothing about the enamel particularly. Up to that time that is what was known. What may seem a little singular to us is the fact that every observer up to within the last five years, ignored, comparatively speaking, everything that Nasmyth did, no credit having been given him by any observer, as far as I have seen. Had his discoveries impressed the dental student of his time as they do the searcher after truth of our "day and generation," we would have known the structure of the teeth to-day very much better than we do. In 1878, a gentleman in New York, Dr. Bodecker, undertook the study of the minute anatomy of the human tooth. His writings upon the subject, which are quite voluminous, may be found in the *Dental Cosmos* in several articles in that and the following year. He gives us drawings as well, from two hundred and fifty to two thousand diameters, one-fifth the size of the diagram which I have the pleasure of showing you here. He shows the reticular structure, which, he asserts, is traversed in every direction by living matter, and in this opinion I heartily concur. This living matter is believed to traverse not only the canaliculi proper, but the net-work which joins the canaliculi together. This frame or net-work is composed of minute tubes formed by the glue-giving basis-substance. In these tubes is a fluid, in the center of which runs the living matter, the fluid serving both as a protector and an insulator. Against the outside of the sheath or tube the lime-salts are deposited, forming the substance of the tooth. The red lines you see running through the dentine, enamel and cement, represent the living matter. The finer white lines which are seen running between the canaliculi and joining them together, known as the reticulum, are believed to contain very minute fibers of the same living matter. As I before stated, the reticular structure of the dentine and enamel were discovered by Alexander Nasmyth, in 1839, but it remained for Dr. C. F. W. Bodecker to make the discovery that the reticulum contained living matter, and that the tooth was as perfectly organized as any other organ of the body. This discovery has rationally explained the peculiar and most acute pain produced by cutting a living tooth. Formerly it was thought that the pain consequent upon cutting with a fine instrument was produced by a vibration of the fluid substance contained in the canaliculi, the vibration being conducted along the canaliculi from the point of contact with the instrument to the pulp. As the canaliculi approach within a short distance of the enamel you will observe that they branch off or divide into two, and sometimes three, smaller canaliculi, in order to accommodate themselves to the finer structure of that substance. At this point, between the enamel and dentine, and all the way down between the cementum and dentine, we have what is called by Bodecker the "interzonal space," instead of the interglobular space, as it was formerly called. The living matter forms a very minute net-work at this space, in many instances so fine that it is impossible distinctly to see it. From this space it runs off into the enamel, to which it is distributed. Passing on through the enamel we see it entering and being distributed to Nasmyth's membrane. You will see in following around this interzonal space, little protoplasmic bodies, extending some distance from it into the enamel. These bodies are apparently irregular in form. They are granular in appearance, the granules being joined together by fibers. These are very small structures of themselves, it is true, but they all represent life. They are the living portion of the tooth.

Going back a few years we find in the *Dental Surgery* published by Tomes in 1873, I think it was, a description of what has since been known as "Tomes' fibers," which consist of short fibers drawn out of the canaliculi of the dentine. The process by which the discovery was made, and the fact established that very delicate fibers existed in the canuliculi, was somewhat as follows: A freshly extracted tooth was taken, its pulp chamber opened to its fullest extent, so that a good hold could be obtained of the pulp. It was then grasped with a pair of small forceps and gradually drawn out of the canals. Upon examination of the surface of the pulp thus pulled out, small fibers were seen extending out from the surface in great numbers, too small, of course, to be seen with the naked eye. I believe the power used was about five hundred. Tomes was, and I believe still is, of the opinion that these fibers were branches of the nerve in the pulp. The cementum of the tooth, as you will observe, covers the root and laps on to the edge of the enamel, the latter passing down on either side underneath the edge of the cementum joined to it. That is the point known as the neck of the tooth, where the gum is, or should be, attached. It is about a sixteenth of an inch long normally, in the mouth. The cement, as I said before, is a substance resembling ordinary compact bone; we find bone corpuscles distributed through its structure, with the numerous off-shoots usually present. Chemically it is almost the same as ordinary compact bone. Not like the alveolar process, which is known as cancellous bone, of which we have a sample in the diagram upon the outside of the root. Following this along we come down to the end of the root of the tooth, the point where the pulp enters. This pulp, as you will observe, consists principally of the blood-vessels and nerves where it enters the root. The nearer we approach to the crown, however, the larger it grows, the excess being made up of myxomatous connective tissue. The bright red lines shown here represent the arteries, the darker red lines the veins, and the white the nerves. You will notice that the nerves beyond a certain point become red, or what is known as non-medulated nerve fibers. It is in this condition that they pass on through and between the odontoblasts (which are seen surrounding the entire pulp) into the dentine. Odontoblasts (tooth-formers) are a row of beautifully organized bodies located on the outside of the pulp, next to the dentine. They have a reticular structure, in the meshes of which water is held until it is displaced by lime-salts. While one row of odontoblasts is being converted into dentine by the deposition of lime-salts, another row is being organized and will take their place in regular order. Thus it is that the pulp is constantly becoming smaller and the dentine thicker. You will observe that the odontoblasts have a perceptible nucleus. It is at that point that the lime-salts are first deposited, and it undoubtedly remains the nucleus after the odontoblast becomes a dentine cell. I have no doubt that the delicate reticulum of the odontoblast still exists in the dentine cell, but so very delicate is it that the highest power of the microscope fails to bring it to view. I want you to remember that the first row of odontoblasts formed is immediately under the enamel, and as each row becomes dentine, another row is formed back, or inside of it, so that the growth of dentine is from without inward, while the growth of the enamel is from within outward. The crown of the tooth with its enamel is all formed before any work is done upon the root. The point where the finishing touches are put on is the extreme end of the root, as far as outward appearances are concerned.

The President has asked whether in cutting a tooth the small protoplasmic bodies seen upon the diagram, projecting from or forming a part of the interzonal space in the substance of the enamel, are to be seen with the naked eye. No, they are altogether too fine for that, but they are readily seen with a power of 500. When the President asked the question I was about to remark that nature is always active, so that work is constantly going on in a healthy tooth in a quiet and imperceptible way. But under the slightest irritation, either from friction, caries or any other source, the odontoblasts become stimulated and the process of dentine forming is renewed with vigor. This is the proper fulfillment of the old adage that "self-protection is the first law of nature." It is under such conditions that we have what is known as secondary dentine deposited. According to Bodecker the living matter that runs into the canaliculi is made up from the sheaths or outer portions of the odontoblasts, and apparently passes between them, instead of out of their ends, as described by Tomes. Outside of the tooth, as you see it stand in the socket, you will observe the periosteum, which is this portion colored purple in the diagram. This membrane is very vascular and exceedingly sensitive. I wish to call attention to the arrangement of its fibers. From their attachment to the root of the tooth, they run in an oblique direction toward the crown, to the point of attachment to the side of the socket. By this wise arrangement you will see that the tooth is always suspended in a sort of sling, so that in biting, the fibers stretch, and as soon as the pressure is relieved they contract again, giving the tooth more or less of an up and down motion in the socket. But for this wise provision of nature, if our teeth were solidly held in the jaws, we would probably become toothless much younger than we now do, as we would break them to pieces in biting any hard substance.

The preceding address was illustrative of the "Diagram of an Incisor Tooth," presented before the Dental Society of the State of New York, which has since been published and may be procured of Prof. Abbott, or ordered of the dental depots. Its possession is necessary to the complete comprehension of the paper.—EDITOR.

